# The impact of antipsychotic adherence on acute care utilization

**DOI:** 10.1186/s12888-023-04558-6

**Published:** 2023-01-24

**Authors:** Anthony J. Perkins, Rezaul Khandker, Ashley Overley, Craig A. Solid, Farid Chekani, Anna Roberts, Paul Dexter, Malaz A. Boustani, Leslie Hulvershorn

**Affiliations:** 1 Department of Biostatistics and Health Data Science, Indiana University School of Medicine, Indianapolis, IN USA; 2 Merck & Co., Inc, Rahway, NJ USA; 3 Eskenazi Health, Indianapolis, IN USA; 4 Department of Psychiatry, Indiana University School of Medicine, Indianapolis, IN USA; 5 Solid Research Group, LLC, St. Paul, MN USA; 6 Regenstrief Institute Inc, Indianapolis, IN USA; 7 Indiana University School of Medicine, Indianapolis, IN USA

**Keywords:** Antipsychotic, Adherence, Acute care utilization, Schizophrenia, Bipolar disorders

## Abstract

**Background:**

Non-adherence to psychotropic medications is common in schizophrenia and bipolar disorders (BDs) leading to adverse outcomes. We examined patterns of antipsychotic use in schizophrenia and BD and their impact on subsequent acute care utilization.

**Methods:**

We used electronic health record (EHR) data of 577 individuals with schizophrenia, 795 with BD, and 618 using antipsychotics without a diagnosis of either illness at two large health systems. We structured three antipsychotics exposure variables: the proportion of days covered (PDC) to measure adherence; medication switch as a new antipsychotic prescription that was different than the initial antipsychotic; and medication stoppage as the lack of an antipsychotic order or fill data in the EHR after the date when the previous supply would have been depleted. Outcome measures included the frequency of inpatient and emergency department (ED) visits up to 12 months after treatment initiation.

**Results:**

Approximately half of the study population were adherent to their antipsychotic medication (a PDC ≥ 0.80): 53.6% of those with schizophrenia, 52.4% of those with BD, and 50.3% of those without either diagnosis. Among schizophrenia patients, 22.5% switched medications and 15.1% stopped therapy. Switching and stopping occurred in 15.8% and 15.1% of BD patients and 7.4% and 20.1% of those without either diagnosis, respectively. Across the three cohorts, non-adherence, switching, and stopping therapy were all associated with increased acute care utilization, even after adjusting for baseline demographics, health insurance, past acute care utilization, and comorbidity.

**Conclusion:**

Non-continuous antipsychotic use is common and associated with high acute care utilization.

**Supplementary Information:**

The online version contains supplementary material available at 10.1186/s12888-023-04558-6.

## Background

Across the globe, more than 65 million people are living with schizophrenia or bipolar disorders (BDs), leading to a substantial individual and societal burden [1]. Several classes of antipsychotics are available to reduce the burden of these two severe mental illnesses [2, 3]. Nevertheless, approximately 56% of patients living with schizophrenia and 44% of those living with BD are non-adherent to their antipsychotics [3]. The WHO defines non-adherence to medications as “a case in which a person’s behavior in taking medication does not correspond with agreed recommendations from health personnel.” [4] For some nonadherent patients, switching antipsychotics may be prudent [5], while among adherent patients, inadequate clinical response or adverse events may lead to switch to alternative antipsychotics [5]. Both non-adherence to and switching of antipsychotics have been linked to acute healthcare utilization in both schizophrenia and BD [6–9].

The choice of measurement of adherence is a long-standing methodological problem. Measures of medication adherence can be classified as (1) objective indicators of medication intake, such as pills counts, electronic monitoring, and serum or plasma levels of antipsychotics, and (2) subjective measures of medication use via patient report or interviewer ratings [10]. Furthermore, psychiatrists may have trouble distinguishing between poor adherence and poor treatment response. Thus, the heterogeneity in study design, exposure and outcome measures, adding to confounding effects and possible biases, and methodological restraints complicate comparability of the results of the current literature.

There is an opportunity to understand the impact of poor adherence on acute care utilization among adult patients living with schizophrenia or BD using real world data captured by electronic health records (EHRs). Currently, the insights available about adherence in these vulnerable individuals are based on data captured by clinical research studies with low external validity, collected with un-scalable, invasive, and expensive data capture methods and tools. To fill this gap in the literature, our study uses the EHRs of two healthcare systems: Indiana University Health, a statewide system that includes 18 hospitals, and Eskenazi Health, a safety net health system with 10 Federally Qualified Health Centers. This study evaluated the impact of poor adherence for antipsychotics on acute care utilization among two cohorts of health system patients living with severe mental illness. The first cohort included adult patients with a diagnosis of schizophrenia and the second cohort included adult patients with BD type I (includes manic or mixed states). The study also used a cohort of adults with no documented diagnosis of schizophrenia and/or BD type I but with a history of continuous antipsychotic use. Those lacking either diagnosis may reflect patients with less severe disease, undiagnosed (or undocumented) disease, or off-label use for other conditions. Our hypothesis was that poor adherence would increase acute care utilization in all three cohorts, but more so in individuals with schizophrenia and BD than in patients without either diagnosis.

## Methods

### Data source and cohort selection

This study has been approved by the Institutional Review Board of Indiana University (IRB no. 2011632512). The Indiana University School of Medicine IRB approved a waiver of informed consent given the retrospective cohort design. All study activities and research methods were performed in accordance with the relevant guidelines and regulations. This study employed EHR data from two health systems in Indiana. The Regenstrief Institute Data Core has access to data from the state’s Health Information Exchange (which includes clinical institutions, labs, and some insurance providers) via the Indiana Network for Patient Care (INPC) database as well as to the EHR data warehouses for Indiana University Health (IUHealth), a statewide system with 18 hospitals as well as outpatient clinics, and Eskenazi Health, the county hospital in Marion County where Indianapolis, Indiana, is located. Available data comes from physician data entry during routine patient care. The data warehouses capture all structured data from patient encounters within these health systems, while the INPC receives specific pieces of clinical data but is not as comprehensive in terms of data elements. The Data Core serves as the honest data broker for access to these data sources for research re-use. Identifiers from the Indiana Biobank are matched to the INPC on a weekly basis, which provides a link between biospecimen samples and all three data sources.

We identified 6,854 patients 18 and older who had at least one 60-day period of continuous antipsychotic use between 2006 and 2018. The earliest date for which antipsychotics were ordered or prescribed was identified as the “first antipsychotic date.” The “treatment date” was set as first day of the 60-day period, unless that period occurred within 6 months of the “first antipsychotic date,” in which case the “treatment date” was set equal to the “first antipsychotic date.”

Patients without a diagnosis of schizophrenia or BD, type I at any time during the study period were categorized as a separate cohort. Patients with diagnoses of schizophrenia and schizoaffective disorder (hereafter referred to together as schizophrenia) (ICD-9 295X or ICD-10 F20 or F25X) or BD (ICD-9 296.0X, 296.1X, 296.4X-6X, 296.7, 296.80, 296.89, or ICD-10 F31X but not F31.81) were categorized as such. We acknowledge that those with schizoaffective disorder are often separated or excluded from studies of schizophrenia, but we feel here that because of the similarities in clinical presentation it was reasonable to group them. Both are classified together in the Diagnostic and Statistical Manual of Mental Disorders as Schizophrenia Spectrum Disorders and share the same core symptoms of psychosis along with a similar age of onset and course of illness, and have long been subject to debate about the validity and clinical utility of any distinction between the diagnoses [11, 12]. Indeed, depending on the quality of the available history and collateral information to describe the course of illness, these diagnoses can be indistinguishable in their presentations at any given time. Interrater reliability of schizoaffective disorder is known to be lower than that of schizophrenia and bipolar disorder [12], meaning that this diagnosis is often subject to change and should be reexamined frequently by clinicians. In the American Psychiatric Association practice guideline, the treatment of schizophrenia [13] includes many studies that included individuals with schizoaffective disorder, noting that these data were rarely analyzed separately, with the result that there is no distinct practice guideline for schizoaffective disorder. Patients with diagnoses for both schizophrenia and BD were categorized based on their most recent diagnosis, as mental health diagnoses routinely change over time within EHRs. If the most recent encounter included diagnoses from both categories, the patients were excluded from our cohort.

### Measures

For each patient we collected age as of the treatment date, race, gender, and insurance status. Using information from EHR records, we identified comorbidities of interest, prior acute care utilization, and previous medication use during the one-year period prior to the treatment date. We defined adherence to antipsychotics using the proportion of days covered (PDC). The PDC is calculated by dividing the number of days the medication is available (from prescription dates) by the number of days in the period of interest. Patients with a PDC ≥ 0.8 were considered to be adherent. Adherence reflected adherence to any antipsychotic medication: if a patient switched medications during the study period, both medications contributed to the PDC calculation. In addition to adherence, we identified medication switches and medication stoppages during the first 6 months of treatment. A medication switch was defined as a new antipsychotic prescription that was different than the initial antipsychotic medication. Medication stoppage was defined as the lack of an antipsychotic medication order or SureScripts dispensing data in the EHR after the date when the previous supply would have been depleted.

To examine acute care utilization after the initiation of antipsychotic therapy, we identified all-cause and mental health-related ED and inpatient admissions during the first six months after the treatment date (a period of observed adherence) and from 6 to 12 months after the treatment date. In addition to overall acute care utilization, ED visits and inpatient admissions were also classified by whether they were for mental health-related care based on a combination of diagnoses, service location, and provider specialty codes.

Given that weight gain has been cited as a risk factor for non-adherence to antipsychotic medications [14–19], we incorporated measures of weight gain into our analyses. Weight gain was calculated as the difference between baseline weight and follow-up weight. Baseline weight was defined as the most recent weight recorded in the EHR prior to the treatment date, not more than 90 days prior to the treatment date. Follow-up weight was defined as the weight recorded in the EHR closest to treatment date plus 90 days, not less than 60 days or more than 120 days after the treatment date. Significant weight gain was described as ≥ 7% increase in weight.

### Statistical analysis

We used Chi-square tests to compare utilization across the three cohorts (schizophrenia, BD, those without either diagnosis) and by different measures of medication adherence and switching. Logistic regression assessed the relationship between utilization during three time points (from treatment date to 6 months post treatment date and from 6 to 12 months post treatment date) and by medication adherence (PDC ≥ 0.8, PDC 0.5 to 0.79, PDC < 0.5) and switching (yes versus no), adjusting for demographic and clinical characteristics. Additional sensitivity analyses were performed using mental health ED visits and inpatient admissions as the outcome. Finally, we performed sensitivity analyses including patients whose weight change could not be calculated.

## Results

We identified 577 individuals with schizophrenia, 795 with BD, and 618 without either diagnosis whose weight change could be calculated at 3 months. The cohort of patients without either diagnosis was older, on average, than those with either schizophrenia or BD, and had a higher prevalence of most comorbid conditions (Table 1). However, alcohol and substance abuse were more common among individuals with schizophrenia or BD than among those without either diagnosis. Approximately half of the study population were adherent to their antipsychotic medication (a PDC ≥ 0.80): 53.6% of those with schizophrenia, 52.4% of those with BD, and 50.3% of those without either diagnosis. Among schizophrenia patients, 22.5% switched medications and 15.1% stopped therapy. Switching and stopping occurred in 15.8% and 15.1% of BD patients and 7.4% and 20.1% of those without either diagnosis, respectively.

**Table 1:** Baseline characteristics, care utilization, and adherence by cohort

	**Neither Bipolar Disorder Nor Schizophrenia (*n* = 618)**	**Bipolar Disorder (*n* = 795)**	**Schizophrenia (*n* = 577)**	***P*-value**
	**n (%)**	**n (%)**	**n (%)**	
Mean Age (SD)	53.9 (16.7)	45.3 (13.7)	44.5 (15.3)	< 0.001
Male	229 (37.1)	247 (31.1)	288 (49.9)	< 0.001
Medicaid Coverage	313 (50.6)	467 (58.7)	302 (52.3)	0.005
Medicare Coverage	234 (37.9)	184 (23.1)	174 (30.2)	< 0.001
Diabetes	193 (31.2)	200 (25.2)	145 (25.1)	0.018
Congestive Heart Failure	72 (11.7)	51 (6.4)	25 (4.3)	< 0.001
Obstructive Sleep Apnea	56 (9.1)	50 (6.3)	21 (3.6)	0.001
Hypertension	354 (57.3)	362 (45.5)	224 (38.8)	< 0.001
Myocardial Infarction	13 (2.1)	7 (0.9)	7 (1.2)	0.135
Cerebrovascular Disease	57 (9.2)	25 (3.1)	17 (3.0)	< 0.001
COPD	105 (17.0)	131 (16.5)	73 (12.7)	0.075
Depression	354 (57.3)	369 (46.4)	196 (34.0)	< 0.001
Cancer	73 (11.8)	41 (5.2)	19 (3.3)	< 0.001
Anxiety	216 (35.0)	380 (47.8)	164 (28.4)	< 0.001
Dementia	63 (10.2)	11 (1.4)	9 (1.6)	< 0.001
Alcohol Abuse	44 (7.1)	121 (15.2)	76 (13.2)	< 0.001
Substance Abuse	192 (31.1)	303 (38.1)	225 (39.0)	0.006
**Any ED Visit**				
0 – 6 Months	249 (40.3)	370 (46.5)	284 (49.2)	0.006
6 -12 Months	198 (32.0)	327 (41.1)	247 (42.8)	< 0.001
Prior Year	313 (50.7)	504 (63.4)	372 (64.5)	< 0.001
**Any Mental Health ED Visit**				
0 – 6 Months	30 (4.8)	91 (11.4)	102 (17.7)	< 0.001
6 -12 Months	18 (2.9)	58 (7.3)	75 (13.0)	< 0.001
**Any Inpatient Admission**				
0 – 6 Months	213 (34.5)	204 (25.7)	166 (28.9)	0.001
6 -12 Months	150 (24.3)	169 (21.3)	113 (19.6)	0.134
Prior Year	258 (41.8)	304 (38.2)	222 (38.5)	0.353
**Any Mental Health Inpatient Admission**				
0 – 6 Months	94 (15.2)	145 (18.2)	135 (23.4)	0.001
6 -12 Months	63 (10.2)	113 (14.2)	88 (15.3)	0.022
**Adherence 0 – 180 days**				0.816
PDC < 0.50	115 (18.6)	138 (17.4)	95 (16.5)	
PDC 0.50 – 0.79	192 (31.1)	240 (30.2)	173 (30.0)	
PDC 0.80 +	311 (50.3)	417 (52.4)	309 (53.6)	
**Adherence/Switch Categories**				< 0.001
No Switch & Adherence < 0.5	56 (9.1)	62 (7.8)	37 (6.4)	
No Switch & Adherence 0.5 – 0.79	129 (20.9)	153 (19.2)	93 (16.1)	
No Switch &Adherence ≥ 0.8	263 (42.6)	335 (42.1)	230 (39.9)	
Switch & Adherence < 0.5	4 (0.7)	18 (2.3)	12 (2.1)	
Switch & Adherence 0.5 – 0.79	14 (2.3)	43 (5.4)	52 (9.0)	
Switch & Adherence ≥ 0.8	28 (4.5)	64 (8.1)	66 (11.4)	
Stop	124 (20.1)	120 (15.1)	87 (15.1)	
**Weight Change**				
7% Weight Gain	96 (15.5)	137 (17.2)	128 (22.2)	0.008

*COPD* chronic obstructive pulmonary disease, *ED* emergency department, *PDC* proportion of days covered, *SD* standard deviation

Comparisons of utilization by the three cohorts are presented in Table 1. While the frequency of ED visits was similar between BD and schizophrenia patients, the latter were more likely to have a mental health-related ED visit during all time periods. Both groups were significantly more likely to have an ED visit (any or mental health-related) than those without either diagnosis. Inpatient admissions were most common in patients without either a schizophrenia or BD diagnosis during the first 6 months after treatment initiation, with no statistically significant differences during the other time periods. However, BD and schizophrenia patients were more likely to have an inpatient admission for mental health than those without either diagnosis. Medication adherence was similar across groups, with approximately half of each cohort with a PDC of 0.80 or higher.

Utilization comparisons by adherence and switching are presented in Table 2. ED visits (any or mental health-related) during each time period were most common among patients who switched and were non-adherent. In contrast, patients who did not switch and remained adherent to therapy had the lowest ED utilization rate. Switching (regardless of adherence) was associated with a higher likelihood of mental health-related ED visits and hospital admissions. Utilization results by switching and adherence separately are presented in Supplemental Tables 1 and 2.

**Table 2:** Comparison of utilization (concurrent and post) with switching/stopping

	**No Switch or Stop**			**Switch**				
	**Adherence < 0.5 (*n* = 155)**	**Adherence 0.5 – 0.79 (*n* = 375)**	**Adherence ≥ 0.8 (*n* = 828)**	**Adherence < 0.5 (*n* = 34)**	**Adherence 0.5 – 0.79 (*n* = 109)**	**Adherence ≥ 0.8 (*n* = 158)**	**Stop (*n* = 331)**	***P*-value**
		**n (%)**	**n (%)**		**n (%)**	**n (%)**	**n (%)**	
**Any ED Visit**								
0 – 6 Months	80 (51.6)	174 (46.4)	321 (38.8)	21 (61.8)	57 (52.3)	74 (46.8)	176 (53.2)	< 0.001
6 -12 Months	72 (46.5)	153 (40.8)	298 (36.0)	15 (44.1)	49 (45.0)	52 (32.9)	133 (40.2)	0.064
Prior year	99 (63.9)	233 (62.1)	446 (53.9)	25 (73.5)	73 (67.0)	106 (67.1)	207 (62.5)	0.001
**Any Mental Health ED Visit**								
0 – 6 Months	13 (8.4)	39 (10.4)	85 (10.3)	11 (32.4)	19 (17.4)	26 (16.5)	30 (9.1)	0.001
6 -12 Months	16 (10.3)	34 (9.1)	51 (6.2)	5 (14.7)	12 (11.0)	16 (10.1)	17 (5.1)	0.032
**Any Inpatient Admission**								
0 – 6 Months	46 (29.7)	120 (32.0)	188 (22.7)	15 (44.1)	42 (38.5)	54 (34.2)	118 (35.6)	< 0.001
6 -12 Months	41 (26.5)	101 (26.9)	152 (18.4)	7 (20.6)	20 (18.4)	29 (18.4)	82 (24.8)	0.009
Prior Year	59 (38.1)	153 (40.8)	297 (35.9)	18 (52.9)	46 (42.2)	61 (38.6)	150 (45.3)	0.050
**Any Mental Health Inpatient Admission**								
0 – 6 Months	30 (19.4)	74 (19.7)	120 (14.5)	10 (29.4)	31 (28.4)	38 (24.0)	71 (21.4)	0.001
6 -12 Months	24 (15.5)	60 (16.0)	92 (11.1)	4 (11.8)	13 (11.9)	20 (12.7)	51 (15.4)	0.235
**Weight Change**								
7% Weight Gain	26 (16.8)	61 (16.3)	133 (16.1)	5 (14.7)	28 (25.7)	49 (31.0)	59 (17.8)	< 0.001

*ED* emergency department

To examine the impact of adherence and switching using logistic regression, adherent patients who did not switch were used as the reference category. These models revealed that during the first 6 months, switching, stopping, and non-adherence were all associated with an increased odds of an (all-cause) inpatient admission (Table 3). The highest odds ratios were associated with switching, which more than doubled the odds of a hospitalization regardless of adherence. Directionally, logistic regression on the likelihood of all-cause ED visit produced similar results, although the magnitude was tempered and not as often statistically significant (Table 4). For example, adherent patients who switched were no more likely to have an ED visit during the first 6 months than the reference group of adherent patients who did not switch.

**Table 3:** Logistic regression model results for any inpatient stay at different time points

	**0 – 6 Months**		**6 -12 Months**	
	**OR (95% CI)**	***P*-value**	**OR (95% CI)**	***P*-value**
**Cohort**				
Psychiatric Control (reference)	1.00		1.00	
Bipolar Disorder	0.66 (0.50, 0.88)	0.004	0.96 (0.71, 1.30)	0.788
Schizophrenia	0.74 (0.55, 1.00)	0.050	0.95 (0.68, 1.33)	0.766
Age	1.01 (1.00, 1.02)	0.228	1.00 (0.99, 1.01)	0.463
Female	0.86 (0.68, 1.08)	0.199	0.96 (0.75, 1.24)	0.762
Medicaid	0.81 (0.59, 1.10)	0.170	0.64 (0.46, 0.90)	0.010
Medicare	1.16 (0.81, 1.67)	0.413	1.10 (0.75, 1.62)	0.614
Diabetes	1.25 (0.97, 1.62)	0.087	1.23 (0.94, 1.62)	0.133
CHF	1.77 (1.17, 2.69)	0.007	2.33 (1.54, 3.53)	< 0.001
OSA	0.93 (0.60, 1.45)	0.760	2.07 (1.34, 3.19)	0.001
Hypertension	0.94 (0.72, 1.21)	0.620	0.99 (0.75, 1.31)	0.927
MI	1.68 (0.71, 3.96)	0.236	0.57 (0.23, 1.41)	0.220
Cerebrovascular Disease	1.33 (0.83, 2.16)	0.241	1.57 (0.97, 2.54)	0.069
COPD	1.24 (0.91, 1.70)	0.167	1.44 (1.05, 1.98)	0.024
Depression	0.90 (0.71, 1.14)	0.372	1.05 (0.81, 1.35)	0.722
Cancer	1.34 (0.89, 2.02)	0.168	1.96 (1.28, 2.98)	0.002
Anxiety	0.99 (0.78, 1.26)	0.924	1.13 (0.87, 1.46)	0.356
Dementia	1.02 (0.59, 1.77)	0.943	0.61 (0.34, 1.09)	0.094
Alcohol Abuse	1.15 (0.82, 1.63)	0.415	1.45 (1.02, 2.07)	0.041
Substance Abuse	1.13 (0.88, 1.45)	0.347	1.09 (0.83, 1.43)	0.545
**Adherence/Switch Categories**				
No Switch & Adherence 0.8 + (reference)	1.00		1.00	
No Switch & Adherence < 0.5	1.34 (0.87, 2.06)	0.189	1.58 (1.02, 2.46)	0.042
No Switch & Adherence 0.5 – 0.79	1.52 (1.12, 2.07)	0.007	1.59 (1.15, 2.20)	0.005
Switch & Adherence < 0.5	2.85 (1.33, 6.15)	0.007	1.29 (0.52, 3.16)	0.581
Switch & Adherence 0.5—0.79	2.49 (1.56, 3.98)	< 0.001	1.04 (0.60, 1.81)	0.885
Switch & Adherence ≥ 0.8	2.13 (1.41, 3.22)	< 0.001	1.06 (0.66, 1.72)	0.805
Stop	1.70 (1.24, 2.33)	0.001	1.23 (0.88, 1.73)	0.233
Any Inpatient Stay Prior Year	4.27 (3.35, 5.44)	< 0.001	2.86 (2.19, 3.74)	< 0.001
Any ED Visit Prior Year	1.96 (1.53, 2.51)	< 0.001	1.69 (1.28, 2.22)	< 0.001
7% Weight Gain	0.99 (0.75, 1.30)	0.917	1.19 (0.89, 1.61)	0.245

*CHF* congestive heart failure, *COPD* chronic obstructive pulmonary disease, *MI* myocardial infarction, *OR* odds ratio, *OSA* obstructive sleep apnea

**Table 4:** Logistic regression model results for any ED Visit at different time points

	**0 – 6 Months**		**6 -12 Months**	
	**OR (95% CI)**	***P*-value**	**OR (95% CI)**	***P*-value**
**Cohort**				
Psychiatric Control (reference)	1.00		1.00	
Bipolar Disorder	1.11 (0.86, 1.43)	0.444	1.20 (0.93, 1.54)	0.153
Schizophrenia	1.28 (0.97, 1.69)	0.078	1.48 (1.13, 1.95)	0.005
Age	0.99 (0.98, 1.00)	0.004	0.99 (0.98, 1.00)	0.010
Female	1.22 (0.99, 1.50)	0.069	1.31 (1.06, 1.61)	0.012
Medicaid	0.88 (0.67, 1.17)	0.387	0.74 (0.57, 0.97)	0.031
Medicare	1.30 (0.93, 1.81)	0.129	0.90 (0.65, 1.25)	0.530
Diabetes	1.08 (0.85, 1.37)	0.538	1.13 (0.89, 1.42)	0.324
CHF	1.49 (0.98, 2.26)	0.065	1.91 (1.27, 2.86)	0.002
OSA	0.80 (0.53, 1.22)	0.309	1.08 (0.71, 1.62)	0.726
Hypertension	1.13 (0.90, 1.43)	0.304	1.08 (0.86, 1.35)	0.534
MI	1.09 (0.46, 2.59)	0.844	0.52 (0.21, 1.28)	0.156
Cerebrovascular Disease	0.82 (0.51, 1.30)	0.389	0.99 (0.63, 1.58)	0.981
COPD	1.15 (0.86, 1.54)	0.358	1.49 (1.12, 1.97)	0.006
Depression	0.93 (0.75, 1.15)	0.482	0.84 (0.68, 1.03)	0.098
Cancer	1.39 (0.93, 2.08)	0.110	1.33 (0.90, 1.96)	0.159
Anxiety	1.10 (0.89, 1.37)	0.377	1.33 (1.08, 1.64)	0.009
Dementia	1.52 (0.88, 2.61)	0.134	0.77 (0.45, 1.34)	0.358
Alcohol Abuse	1.13 (0.82, 1.56)	0.454	0.99 (0.73, 1.35)	0.946
Substance Abuse	1.25 (0.99, 1.56)	0.056	1.31 (1.05, 1.63)	0.017
**Adherence/Switch Categories**				
No Switch & Adherence 0.8 + (reference)	1.00		1.00	
No Switch & Adherence < 0.5	1.58 (1.07, 2.32)	0.020	1.41 (0.97, 2.04)	0.073
No Switch & Adherence 0.5 – 0.79	1.25 (0.95, 1.65)	0.108	1.17 (0.89, 1.53)	0.261
Switch & Adherence < 0.5	2.04 (0.95, 4.40)	0.069	1.19 (0.57, 2.46)	0.648
Switch & Adherence 0.5—0.79	1.43 (0.92, 2.22)	0.115	1.22 (0.79, 1.88)	0.368
Switch & Adherence ≥ 0.8	1.20 (0.82, 1.75)	0.351	0.75 (0.51, 1.10)	0.144
Stop	1.63 (1.22, 2.16)	0.001	1.04 (0.79, 1.38)	0.780
Any Inpatient Stay Prior Year	1.80 (1.44, 2.26)	< 0.001	1.07 (0.86, 1.34)	0.556
Any ED Visit Prior Year	4.31 (3.47, 5.35)	< 0.001	2.89 (2.33, 3.59)	< 0.001
7% Weight Gain	0.91 (0.70, 1.18)	0.463	0.68 (0.52, 0.87)	0.003

*CHF* congestive heart failure, *COPD* chronic obstructive pulmonary disease, *MI* myocardial infarction, *OR* odds ratio, *OSA* obstructive sleep apnea

Logistic regression results for mental health-related inpatient admissions and ED visits are presented in supplemental Tables 3 and 4. While the results for mental health-related inpatient admissions are congruent with those for all-cause admissions, results for mental-health related ED visits differ substantially from results for all-cause ED visits. Specifically, non-adherent patients (PDC < 0.5) who switched were the only patients who were more likely to have a mental health-related ED visit than the reference group. Sensitivity analyses using all patients regardless of available data to calculate weight change produced similar results (Supplemental Tables 5 and 6).

## Discussion

In the current study, more than half of the study population was non-adherent (PDC < 0.8) to their antipsychotic medication, and that non-adherence, switching, or stopping antipsychotic therapy was associated with higher acute care utilization, both overall and for mental health-related care. This effect was most notable when looking at utilization during the same time frame as adherence. When looking at the association of adherence and switching with future acute care utilization, the association was weaker. This may be due to some adherent patients becoming non-adherent in the subsequent 6 months. These results held for all three cohorts examined. The neither diagnosis group could represent patients with either undiagnosed (or uncoded) illness or who were using antipsychotics “off-label” for another condition. It is notable that this group was approximately ten years older with a higher comorbidity burden and significantly more documented dementia than either the schizophrenia or BD cohort. Off-label use may also explain why the cohort with neither diagnosis had less switching and more stoppage than the other two cohorts.

Other studies have also linked antipsychotic adherence and switching to healthcare utilization. Noordsy et al. studied California Medicaid patients with schizophrenia and found that those who switched antipsychotics were significantly more likely to have an inpatient hospitalization within six months when compared with those who had continuous treatment [7]. Several outpatient services, like ED visits and physician visits, were higher among those who switched or stopped medication compared with those with continuous treatment, although the authors note that those who switched had higher baseline comorbidity burden and higher rates of prior psychiatric ED visits. In our study population, the association between continued antipsychotic use and utilization remained after adjusting for baseline comorbidities. Of note, our study examined utilization that was both concurrent and subsequent to the period where adherence was measured and found that the association between adherence and utilization was stronger during the concurrent period.

Although we observed higher rates of both all-cause and mental health-related acute care utilization for those without continuous adherent therapy, other studies have reached different conclusions. Joe et al. studied individuals with schizophrenia in South Korea and observed that compared to adherent patients, non-adherent patients had less psychiatric-related utilization, but more non-psychiatric-related utilization and higher overall healthcare costs. [6] The authors hypothesize that “non-compliance” with medications may signal a general behavior of avoiding psychiatric-related healthcare. Regardless, the increase in all-cause utilization is congruent with our results.

A primary strength of this analysis is the large sample of patients and the use of EHR data to establish patterns of antipsychotic use. However, the results should be viewed in light of some limitations. First, PDC is an inexact surrogate for medication adherence and therefore we cannot know for certain whether study patients are truly adherent or not. It is possible that EHR data can misclassify adherence if, for instance, medication fill data was unavailable from the medical record. Further, a cut-off of 0.80, while a common threshold to establish adherence, has not been empirically established as an appropriate threshold within this population. Additionally, this study does not consider antipsychotic dose or changes in dosing, which could impact the results.

Despite these limitations, our results provide further evidence of the association between non-continuous antipsychotic medication use and increased acute care utilization.

## Supplementary Information

**Additional file 1.**

## Data Availability

The dataset supporting the conclusions of this article are available on request.
