# Supplementary material for: The impact of antipsychotic adherence on acute care utilization
Source: BMC Psychiatry. 2023 Jan 24;23:64. doi: 10.1186/s12888-023-04558-6 (PMC9875466; doi:10.1186/s12888-023-04558-6)
Supplement: Supplementary file 1 — Additional file 1. [file 12888_2023_4558_MOESM1_ESM.docx]

**Supplemental Table 1. Comparison of utilization (concurrent and post) with switching/stopping**

|  | **No Switch or Stop**  **(n=1358)** | **Switch**  **(n=301)** | **Stop**  **(n=331)** | **P-value** |
| --- | --- | --- | --- | --- |
|  | **n (%)** | **n (%)** | **n (%)** |  |
| **Any ED Visit** |  |  |  |  |
| 0 – 6 Months | 575 (42.3) | 152 (50.5) | 176 (53.2) | <0.001 |
| 6 -12 Months | 523 (38.5) | 116 (38.5) | 133 (40.2) | 0.851 |
| Prior Year | 778 (57.3) | 204 (67.8) | 207 (62.5) | 0.002 |
| **Any Mental Health ED Visit** |  |  |  |  |
| 0 – 6 Months | 137 (10.1) | 56 (18.6) | 30 (9.1) | <0.001 |
| 6 -12 Months | 101 (7.4) | 33 (11.0) | 17 (5.1) | 0.021 |
| **Any Inpatient Admission** |  |  |  |  |
| 0 – 6 Months | 354 (26.1) | 111 (36.9) | 118 (35.6) | <0.001 |
| 6 -12 Months | 294 (21.7) | 56 (18.6) | 82 (24.8) | 0.171 |
| Prior Year | 509 (37.5) | 125 (41.5) | 150 (45.3) | 0.023 |
| **Any Mental Health Inpatient Admission** |  |  |  |  |
| 0 – 6 Months | 224 (16.5) | 79 (26.3) | 71 (21.4) | <0.001 |
| 6 -12 Months | 176 (13.0) | 37 (12.3) | 51 (15.4) | 0.432 |
| **Weight Change** |  |  |  |  |
| 7% Weight Gain | 220 (16.2) | 82 (27.2) | 59 (17.8) | <0.001 |

**Supplemental Table 2. Comparison of utilization (concurrent and post) with adherence from 0 to 180 days**

|  | **Adherence**  **0 – 0.49**  **(n=348)** | **Adherence**  **0.5 – 0.79**  **(n=303)** | **Adherence**  **0.8 – 1.0**  **(n=1037)** | **P-value** |
| --- | --- | --- | --- | --- |
|  | **n (%)** | **n (%)** | **n (%)** |  |
| **Any ED Visit** |  |  |  |  |
| 0 – 6 Months | 182 (52.3) | 303 (50.1) | 418 (40.3) | <0.001 |
| 6 -12 Months | 145 (41.7) | 257 (42.5) | 370 (35.7) | 0.012 |
| Prior Year | 222 (63.8) | 382 (63.1) | 585 (56.4) | 0.007 |
| **Any Mental Health ED Visit** |  |  |  |  |
| 0 – 6 Months | 38 (10.9) | 69 (11.4) | 116 (11.2) | 0.974 |
| 6 -12 Months | 26 (7.5) | 54 (8.9) | 71 (6.8) | 0.307 |
| **Any Inpatient Admission** |  |  |  |  |
| 0 – 6 Months | 113 (32.5) | 210 (34.7) | 260 (25.1) | <0.001 |
| 6 -12 Months | 78 (22.4) | 156 (25.8) | 198 (19.1) | 0.006 |
| Prior Year | 150 (43.1) | 253 (41.8) | 381 (36.7) | 0.028 |
| **Any Mental Health Inpatient Admission** |  |  |  |  |
| 0 – 6 Months | 70 (20.1) | 134 (22.2) | 170 (16.4) | 0.012 |
| 6 - 12 Months | 47 (13.5) | 94 (15.5) | 123 (11.9) | 0.105 |
| **Weight Change** |  |  |  |  |
| 7% Weight Gain | 62 (17.8) | 109 (18.0) | 190 (18.3) | 0.973 |

**Supplemental Table 3. Logistic Regression Model Results for Any Behavioral Health Inpatient Stay at different time points**

|  | **0 – 6 Months** | | **6 -12 Months** | |
| --- | --- | --- | --- | --- |
|  | **OR (95% CI)** | **P-value** | **OR (95% CI)** | **P-value** |
| **Cohort** |  |  |  |  |
| Psychiatric Control (reference) | 1.00 |  | 1.00 |  |
| Bipolar Disorder | 1.48 (1.06, 2.08) | 0.023 | 1.81 (1.23, 2.66) | 0.003 |
| Schizophrenia | 2.07 (1.45, 2.95) | <0.001 | 2.16 (1.43, 3.25) | <0.001 |
| Age | 1.00 (0.99, 1.01) | 0.539 | 1.00 (0.98, 1.01) | 0.546 |
| Female | 0.99 (0.76, 1.28) | 0.923 | 1.01 (0.75, 1.36) | 0.943 |
| Medicaid | 0.81 (0.57, 1.15) | 0.242 | 0.74 (0.49, 1.10) | 0.135 |
| Medicare | 1.31 (0.88, 1.96) | 0.183 | 1.30 (0.83, 2.03) | 0.255 |
| Diabetes | 1.06 (0.80, 1.40) | 0.708 | 1.58 (1.15, 2.16) | 0.004 |
| CHF | 1.66 (1.09, 2.53) | 0.017 | 2.54 (1.63, 3.95) | <0.001 |
| OSA | 0.78 (0.48, 1.27) | 0.325 | 1.40 (0.86, 2.28) | 0.180 |
| Hypertension | 1.21 (0.90, 1.62) | 0.211 | 0.81 (0.58, 1.14) | 0.227 |
| MI | 0.79 (0.32, 1.97) | 0.618 | 0.69 (0.24, 2.00) | 0.499 |
| Cerebrovascular Disease | 1.40 (0.86, 2.29) | 0.180 | 1.73 (1.02, 2.93) | 0.043 |
| COPD | 1.49 (1.07, 2.07) | 0.018 | 1.22 (0.84, 1.77) | 0.297 |
| Depression | 0.92 (0.70, 1.19) | 0.507 | 1.04 (0.77, 1.39) | 0.814 |
| Cancer | 1.44 (0.92, 2.26) | 0.114 | 1.54 (0.93, 2.53) | 0.093 |
| Anxiety | 1.06 (0.81, 1.38) | 0.683 | 1.20 (0.89, 1.62) | 0.239 |
| Dementia | 1.22 (0.68, 2.17) | 0.506 | 0.86 (0.43, 1.71) | 0.671 |
| Alcohol Abuse | 1.04 (0.71, 1.52) | 0.838 | 1.17 (0.77, 1.77) | 0.474 |
| Substance Abuse | 1.06 (0.80, 1.40) | 0.679 | 1.14 (0.83, 1.56) | 0.434 |
| **Adherence/Switch Categories** |  |  |  |  |
| No Switch & Adherence 0.8+ (reference) | 1.00 |  | 1.00 |  |
| No Switch & Adherence < 0.5 | 1.39 (0.86, 2.26) | 0.182 | 1.46 (0.86, 2.46) | 0.162 |
| No Switch & Adherence 0.5 – 0.79 | 1.41 (0.99, 1.99) | 0.056 | 1.49 (1.02, 2.17) | 0.041 |
| Switch & Adherence < 0.5 | 2.22 (0.98, 5.04) | 0.056 | 0.99 (0.33, 3.00) | 0.991 |
| Switch & Adherence 0.5 - 0.79 | 2.34 (1.42, 3.85) | 0.001 | 1.04 (0.54, 1.99) | 0.909 |
| Switch & Adherence ≥ 0.8 | 1.86 (1.18, 2.93) | 0.007 | 1.13 (0.65, 1.96) | 0.671 |
| Stop | 1.42 (1.00, 2.03) | 0.052 | 1.30 (0.88, 1.94) | 0.191 |
|  |  |  |  |  |
| Any Inpatient Stay Prior Year | 3.88 (2.93, 5.14) | <0.001 | 2.58 (1.86, 3.56) | <0.001 |
| Any ED Visit Prior Year | 1.49 (1.12, 1.99) | 0.006 | 1.59 (1.14, 2.22) | 0.007 |
| 7% Weight Gain | 1.12 (0.83, 1.51) | 0.473 | 1.08 (0.76, 1.53) | 0.669 |

**Supplemental Table 4. Logistic Regression Model Results for Any Behavioral Health ED Visit at different time points**

|  | **0 – 6 Months** | | **6 -12 Months** | |
| --- | --- | --- | --- | --- |
|  | **OR (95% CI)** | **P-value** | **OR (95% CI)** | **P-value** |
| **Cohort** |  |  |  |  |
| Psychiatric Control (reference) | 1.00 |  | 1.00 |  |
| Bipolar Disorder | 1.99 (1.25, 3.17) | 0.004 | 2.15 (1.21, 3.82) | 0.009 |
| Schizophrenia | 3.16 (1.97, 5.08) | <0.001 | 4.17 (2.35, 7.38) | <0.001 |
| Age | 0.98 (0.97, 1.00) | 0.007 | 0.98 (0.97, 1.00) | 0.024 |
| Female | 0.77 (0.56, 1.05) | 0.095 | 0.75 (0.52, 1.08) | 0.128 |
| Medicaid | 0.62 (0.42, 0.91) | 0.015 | 1.01 (0.62, 1.63) | 0.972 |
| Medicare | 1.08 (0.68, 1.69) | 0.756 | 1.53 (0.88, 2.67) | 0.136 |
| Diabetes | 0.74 (0.51, 1.07) | 0.107 | 0.94 (0.61, 1.45) | 0.781 |
| CHF | 1.13 (0.62, 2.07) | 0.683 | 1.24 (0.62, 2.50) | 0.541 |
| OSA | 1.11 (0.59, 2.07) | 0.752 | 1.13 (0.55, 2.30) | 0.747 |
| Hypertension | 1.21 (0.85, 1.72) | 0.297 | 0.91 (0.60, 1.38) | 0.667 |
| MI | 0.46 (0.10, 2.12) | 0.318 | 1.19 (0.31, 4.59) | 0.802 |
| Cerebrovascular Disease | 0.73 (0.34, 1.57) | 0.422 | 0.91 (0.36, 2.26) | 0.832 |
| COPD | 1.18 (0.76, 1.82) | 0.457 | 1.02 (0.60, 1.73) | 0.940 |
| Depression | 0.98 (0.72, 1.35) | 0.913 | 1.37 (0.95, 1.99) | 0.096 |
| Cancer | 1.18 (0.64, 2.17) | 0.607 | 1.90 (0.99, 3.62) | 0.052 |
| Anxiety | 1.06 (0.76, 1.46) | 0.742 | 1.09 (0.75, 1.60) | 0.641 |
| Dementia | 1.46 (0.63, 3.42) | 0.378 | 0.22 (0.03, 1.68) | 0.144 |
| Alcohol Abuse | 1.29 (0.86, 1.95) | 0.224 | 1.26 (0.77, 2.08) | 0.360 |
| Substance Abuse | 1.25 (0.90, 1.73) | 0.188 | 1.04 (0.70, 1.53) | 0.847 |
| **Adherence/Switch Categories** |  |  |  |  |
| No Switch & Adherence 0.8+ (reference) | 1.00 |  | 1.00 |  |
| No Switch & Adherence < 0.5 | 0.79 (0.42, 1.49) | 0.462 | 1.86 (1.00, 3.47) | 0.050 |
| No Switch & Adherence 0.5 – 0.79 | 0.96 (0.63, 1.47) | 0.866 | 1.52 (0.94, 2.44) | 0.085 |
| Switch & Adherence < 0.5 | 3.23 (1.44, 7.26) | 0.005 | 1.86 (0.66, 5.20) | 0.240 |
| Switch & Adherence 0.5 - 0.79 | 1.24 (0.69, 2.22) | 0.472 | 1.28 (0.64, 2.57) | 0.492 |
| Switch & Adherence ≥ 0.8 | 1.31 (0.79, 2.19) | 0.296 | 1.31 (0.71, 2.43) | 0.392 |
| Stop | 0.75 (0.48, 1.19) | 0.226 | 0.78 (0.44, 1.40) | 0.405 |
|  |  |  |  |  |
| Any Inpatient Stay Prior Year | 2.00 (1.44, 2.78) | <0.001 | 1.69 (1.15, 2.48) | 0.008 |
| Any ED Visit Prior Year | 2.56 (1.74, 3.78) | <0.001 | 2.46 (1.56, 3.87) | <0.001 |
| 7% Weight Gain | 1.02 (0.72, 1.46) | 0.898 | 0.82 (0.53, 1.28) | 0.384 |

**Supplemental Table 5. Logistic Regression Model Results for Any Inpatient Stay at different time points using full sample**

|  | **0 – 6 Months** | | **6 -12 Months** | |
| --- | --- | --- | --- | --- |
|  | **OR (95% CI)** | **P-value** | **OR (95% CI)** | **P-value** |
| **Cohort** |  |  |  |  |
| Psychiatric Control (reference) | 1.00 |  | 1.00 |  |
| Bipolar Disorder | 0.91 (0.77, 1.07) | 0.244 | 1.02 (0.86, 1.20) | 0.844 |
| Schizophrenia | 0.98 (0.83, 1.16) | 0.803 | 0.93 (0.79, 1.11) | 0.434 |
| Age | 1.00 (1.00, 1.01) | 0.118 | 1.01 (1.00, 1.01) | 0.015 |
| Female | 0.81 (0.71, 0.92) | 0.001 | 1.03 (0.90, 1.18) | 0.644 |
| Medicaid | 0.91 (0.77, 1.08) | 0.295 | 0.90 (0.75, 1.08) | 0.248 |
| Medicare | 1.33 (1.09, 1.62) | 0.005 | 1.55 (1.27, 1.90) | <0.001 |
| Diabetes | 1.38 (1.19, 1.61) | <0.001 | 1.11 (0.95, 1.30) | 0.184 |
| CHF | 1.51 (1.21, 1.89) | <0.001 | 1.75 (1.41, 2.19) | <0.001 |
| OSA | 0.94 (0.76, 1.15) | 0.523 | 1.38 (1.13, 1.68) | 0.001 |
| Hypertension | 0.97 (0.83, 1.13) | 0.675 | 0.95 (0.81, 1.12) | 0.545 |
| MI | 0.93 (0.67, 1.30) | 0.672 | 1.07 (0.76, 1.49) | 0.712 |
| Cerebrovascular Disease | 1.25 (1.01, 1.54) | 0.041 | 1.40 (1.13, 1.73) | 0.002 |
| COPD | 1.37 (1.15, 1.63) | 0.001 | 1.36 (1.14, 1.62) | 0.001 |
| Depression | 0.92 (0.80, 1.05) | 0.222 | 0.86 (0.74, 0.99) | 0.034 |
| Cancer | 1.10 (0.92, 1.32) | 0.302 | 0.95 (0.79, 1.15) | 0.601 |
| Anxiety | 0.90 (0.79, 1.03) | 0.135 | 0.90 (0.78, 1.04) | 0.159 |
| Dementia | 1.03 (0.79, 1.35) | 0.833 | 0.78 (0.59, 1.03) | 0.083 |
| Alcohol Abuse | 0.99 (0.84, 1.17) | 0.899 | 1.14 (0.96, 1.35) | 0.143 |
| Substance Abuse | 0.96 (0.84, 1.11) | 0.617 | 1.02 (0.88, 1.18) | 0.809 |
| **Adherence/Switch Categories** |  |  |  |  |
| No Switch & Adherence 0.8+ (reference) | 1.00 |  | 1.00 |  |
| No Switch & Adherence < 0.5 | 1.17 (0.92, 1.48) | 0.201 | 1.26 (1.00, 1.59) | 0.047 |
| No Switch & Adherence 0.5 – 0.79 | 1.40 (1.17, 1.66) | <0.001 | 1.23 (1.04, 1.46) | 0.019 |
| Switch & Adherence < 0.5 | 2.37 (1.57, 3.56) | <0.001 | 1.19 (0.76, 1.88) | 0.449 |
| Switch & Adherence 0.5 - 0.79 | 2.43 (1.85, 3.20) | <0.001 | 0.80 (0.57, 1.12) | 0.197 |
| Switch & Adherence ≥ 0.8 | 2.25 (1.78, 2.84) | <0.001 | 1.05 (0.81, 1.37) | 0.715 |
| Stop | 1.34 (1.12, 1.59) | 0.001 | 0.85 (0.70, 1.02) | 0.086 |
|  |  |  |  |  |
| Any Inpatient Stay Prior Year | 3.12 (2.74, 3.55) | <0.001 | 2.19 (1.92, 2.51) | <0.001 |
| Any ED Visit Prior Year | 1.65 (1.44, 1.89) | <0.001 | 1.38 (1.20, 1.58) | <0.001 |

**Supplemental Table 6. Logistic Regression Model Results for Any ED Visit at different time points using full sample**

|  | **0 – 6 Months** | | **6 -12 Months** | |
| --- | --- | --- | --- | --- |
|  | **OR (95% CI)** | **P-value** | **OR (95% CI)** | **P-value** |
| **Cohort** |  |  |  |  |
| Psychiatric Control (reference) | 1.00 |  | 1.00 |  |
| Bipolar Disorder | 1.18 (1.03, 1.37) | 0.022 | 1.41 (1.22, 1.62) | <0.001 |
| Schizophrenia | 1.13 (0.98, 1.31) | 0.098 | 1.51 (1.30, 1.74) | <0.001 |
| Age | 0.99 (0.99, 1.00) | <0.001 | 0.99 (0.99, 1.00) | 0.001 |
| Female | 1.06 (0.95, 1.19) | 0.319 | 1.17 (1.04, 1.30) | 0.008 |
| Medicaid | 0.88 (0.77, 1.02) | 0.089 | 0.85 (0.74, 0.98) | 0.025 |
| Medicare | 0.96 (0.81, 1.14) | 0.650 | 1.11 (0.93, 1.31) | 0.251 |
| Diabetes | 1.28 (1.11, 1.47) | 0.001 | 1.13 (0.99, 1.30) | 0.078 |
| CHF | 1.33 (1.07, 1.65) | 0.010 | 1.41 (1.14, 1.75) | 0.002 |
| OSA | 0.91 (0.76, 1.09) | 0.282 | 1.10 (0.92, 1.31) | 0.288 |
| Hypertension | 1.10 (0.96, 1.25) | 0.175 | 0.99 (0.87, 1.12) | 0.825 |
| MI | 1.12 (0.82, 1.54) | 0.482 | 0.90 (0.65, 1.23) | 0.504 |
| Cerebrovascular Disease | 1.03 (0.84, 1.26) | 0.793 | 1.17 (0.96, 1.42) | 0.121 |
| COPD | 1.15 (0.98, 1.35) | 0.091 | 1.27 (1.08, 1.49) | 0.004 |
| Depression | 1.02 (0.90, 1.15) | 0.797 | 0.99 (0.88, 1.12) | 0.869 |
| Cancer | 1.10 (0.94, 1.30) | 0.236 | 1.24 (1.06, 1.45) | 0.008 |
| Anxiety | 1.07 (0.95, 1.21) | 0.244 | 1.12 (1.00, 1.26) | 0.050 |
| Dementia | 1.11 (0.85, 1.43) | 0.453 | 1.05 (0.81, 1.36) | 0.718 |
| Alcohol Abuse | 1.15 (1.00, 1.33) | 0.053 | 1.05 (0.91, 1.21) | 0.479 |
| Substance Abuse | 1.24 (1.10, 1.40) | 0.001 | 1.25 (1.11, 1.41) | <0.001 |
| **Adherence/Switch Categories** |  |  |  |  |
| No Switch & Adherence 0.8+ (reference) | 1.00 |  | 1.00 |  |
| No Switch & Adherence < 0.5 | 1.20 (0.98, 1.47) | 0.080 | 1.24 (1.02, 1.51) | 0.034 |
| No Switch & Adherence 0.5 – 0.79 | 1.31 (1.13, 1.52) | <0.001 | 1.14 (0.99, 1.32) | 0.076 |
| Switch & Adherence < 0.5 | 2.12 (1.44, 3.12) | <0.001 | 1.39 (0.95, 2.04) | 0.086 |
| Switch & Adherence 0.5 - 0.79 | 1.79 (1.39, 2.30) | <0.001 | 1.01 (0.79, 1.31) | 0.914 |
| Switch & Adherence ≥ 0.8 | 1.47 (1.19, 1.82) | <0.001 | 1.01 (0.81, 1.25) | 0.938 |
| Stop | 1.24 (1.07, 1.45) | 0.004 | 0.89 (0.77, 1.04) | 0.143 |
|  |  |  |  |  |
| Any Inpatient Admission Prior Year | 1.51 (1.35, 1.70) | <0.001 | 1.14 (1.01, 1.28) | 0.028 |
| Any ED Visit Prior Year | 3.08 (2.75, 3.46) | <0.001 | 2.42 (2.16, 2.71) | <0.001 |
